# Integrated Mendelian Randomization and Single‐Cell RNA Sequencing Analyses Reveal Lactate Metabolism as a Key Pathway in COVID‐19‐Induced Pulmonary Fibrosis

**DOI:** 10.1155/carj/1049336

**Published:** 2026-01-11

**Authors:** Xin Zhang, Liping Jia, Tuersun Yeziya, Shuyan Yang, Miaomiao Chen, Yan Mo, Xia Tong, Lanlan Zhang

**Affiliations:** ^1^ Department of Gastroenterology, The First People’s Hospital of Shuangliu District (West China Airport Hospital of Sichuan University), Chengdu, 610200, China; ^2^ Key Laboratory of Birth Defects and Related Diseases of Women and Children of MOE, State Key Laboratory of Biotherapy, West China Second University Hospital, Sichuan University, Chengdu, 610064, China, scu.edu.cn; ^3^ Department of Internal Medicine, West China Second University Hospital, Sichuan University, Chengdu, Sichuan, China, scu.edu.cn; ^4^ Laboratory of Obstetric and Gynecologic and Pediatric Diseases and Birth Defects of Ministry of Education, Sichuan University, Chengdu, Sichuan, China, scu.edu.cn; ^5^ Department of Respiratory and Critical Care Medicine, Tianjin Chest Hospital, Tianjin, 300010, China, chesthospital.com; ^6^ Department of Neurology Medicine, The Aviation Industry Corporation of China (AVIC) 363 Hospital, Chengdu, China; ^7^ Department of Pulmonary and Critical Care Medicine, State Key Laboratory of Respiratory Health and Multimorbidity, West China Hospital, Sichuan University, Chengdu, 610064, China, scu.edu.cn

**Keywords:** COVID-19, lactate metabolism, Mendelian randomization (MR), pulmonary fibrosis, single-cell RNA sequencing (scRNA), SLC16A4

## Abstract

**Background:**

The COVID‐19 pandemic has led to a variety of long‐term complications, with COVID‐19‐induced idiopathic pulmonary fibrosis (IPF) becoming a major concern. However, the underlying mechanisms, effective therapeutic strategies, and long‐term prognosis of COVID‐19‐related pulmonary fibrosis remain unclear.

**Methods:**

This study utilized Mendelian randomization (MR) analysis and single‐cell RNA sequencing (scRNA‐seq) to systematically investigate the molecular mechanisms underlying COVID‐19‐induced pulmonary fibrosis. MR analysis was conducted to assess causal relationships, while scRNA‐seq provided detailed insights into the cellular and molecular processes involved in fibrosis.

**Results:**

MR analysis revealed a significant association between COVID‐19 infection and the development of IPF (OR = 1.15, 95% CI = 1.05–1.25, *p* = 0.001), whereas the reverse causality—IPF increasing the risk of COVID‐19 infection—was not significant. Mediation analysis identified lactate metabolism as a crucial intermediary pathway in COVID‐19‐induced IPF (OR = 1.30, 95% CI = 1.09–1.55, *p* = 0.003). scRNA‐seq confirmed the central role of lactate metabolism in pulmonary fibrosis, particularly in lung epithelial cells. The key lactate transport gene, SLC16A4, was found to play a significant role in the progression of fibrosis. Additionally, cellular interaction analysis revealed that lung epithelial cells interacted with fibroblasts via the PDGFC–PDGFRA signaling axis, promoting fibrosis.

**Conclusion:**

This study uncovers a critical mechanism by which COVID‐19 promotes pulmonary fibrosis through the regulation of lactate metabolism in lung epithelial cells, with SLC16A4 playing a pivotal role. These findings highlight the potential of targeting this metabolic pathway as a therapeutic approach for pulmonary fibrosis, offering new directions for future antifibrotic treatment strategies.

## 1. Background

COVID‐19 infection has been shown to lead to a variety of long‐term complications, with pulmonary fibrosis being one of the most serious outcomes. Pulmonary fibrosis, especially in critically ill COVID‐19 patients, is a common and persistent complication, and its long‐term risk has been the focus of research [1–4]. A growing number of studies have revealed that diffuse alveolar damage (DAD) is a common pathological manifestation in patients with COVID‐19 and that the fibrotic process in the lungs continues in some patients even after recovery [5–7]. It has been found that more than 50% of patients with severe COVID‐19 continue to exhibit imaging abnormalities 6 months after infection, and some cases continue to show a decline in lung function even after one year [8, 9].

COVID‐19‐induced pulmonary fibrosis is a multifactorial‐driven pathological process involving immune response, metabolic dysregulation, and excitation of fibrotic signaling pathways. SARS‐CoV‐2 enters the cells by binding to angiotensin‐converting enzyme 2 (ACE2) receptors on the surface of lung epithelial cells, which subsequently activates fibrotic signaling pathways, such as transforming growth factor‐β (TGF‐β). This activation leads to proliferation of fibroblasts and excessive deposition of extracellular matrix (ECM), ultimately triggering fibrosis [1, 9–11]. Although the importance of these signaling pathways has been extensively studied, the specific role of metabolic regulation, particularly lactate metabolism, in fibrosis has not been fully explored.

In recent years, the role of lactate metabolism in pulmonary fibrosis has received increasing attention. Studies have shown that lactate is not only a by‐product of cellular metabolism but also participates in the regulation of the fibrotic process. In idiopathic pulmonary fibrosis (IPF), lactate levels are significantly elevated, and lactate accumulation further promotes fibrosis by altering the local pH environment, activating the TGF‐β signaling pathway, and promoting myofibroblast proliferation and ECM deposition [12, 13]. This finding provides a new perspective on how metabolic dysregulation affects COVID‐19‐associated pulmonary fibrosis, but the specific regulatory mechanism remains unclear.

We aimed to conduct the first MR study to our knowledge to assess the causal relationship and mediating pathways between COVID‐19 and IPF. Subsequently, the metabolic regulatory mechanisms of COVID‐19‐induced pulmonary fibrosis were also systematically explored in conjunction with single‐cell transcriptome sequencing analyses, particularly the role of lactate metabolism in the process. Finally, we conducted in vitro functional validation using pseudovirus‐treated alveolar epithelial cells and fibroblast coculture assays, confirming that lactate accumulation in epithelial cells can directly trigger fibroblast activation through defined signaling interactions. Together, these multilevel analyses establish lactate metabolism as a mechanistically important driver of COVID‐19–associated pulmonary fibrosis.

## 2. Methodologies

### 2.1. Data Sources

Data for COVID‐19 were derived from a genome‐wide association study (GWAS) including 1,388,342 participants of European ancestry. Metabolite data were derived from 1400 metabolite analyses of 8299 Canadian Longitudinal Study of Aging (CLSA) cohort individuals; IPF data were derived from the FinnGen Consortium study comprising 409,798 participants (2189 in the case group and 407,609 in the control group). In addition, we downloaded single‐cell RNA sequencing (scRNA‐seq) datasets from the Gene Expression Omnibus (GEO) database of lung tissues from COVID‐19 patients, including GSE171524, GSE171668, GSE149878, GSE161382, and GSE163919 [14–17].

### 2.2. Mendelian Randomization Analysis

**Figure 1:**
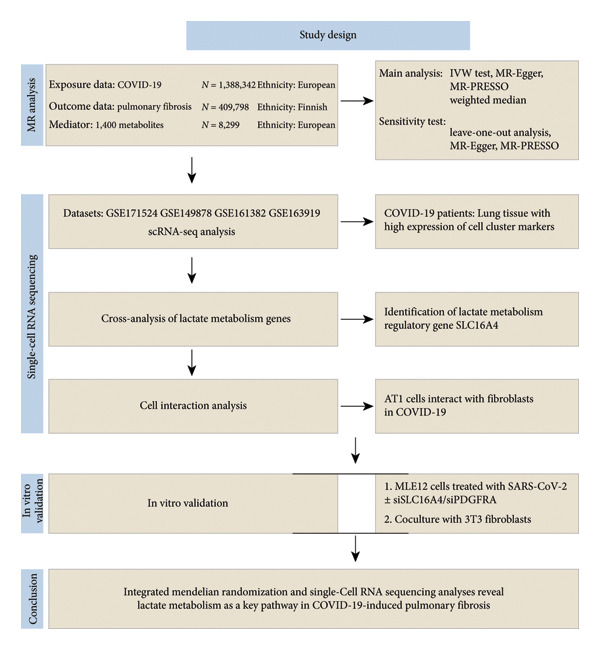
Flowchart of the study.

In this study, the causal relationship between COVID‐19 and IPF was explored using MR analysis (Figure 1). The inverse variance weighting (IVW) method, along with the weighted median, was applied using the “TwoSampleMR” (Version 0.5.7) and “MR‐PRESSO” (Version 1.0) packages in *R* software (Version 4.3.1). MR‐Egger, as well as simple and weighted models, was utilized in a two‐sample MR framework to evaluate the causal effect of COVID‐19 on IPF and to estimate the odds ratio (OR) and 95% confidence interval (CI). Additionally, reverse MR analysis was conducted to examine the potential causal effect of IPF on COVID‐19. The statistical significance threshold was set at *p* < 0.05, and sensitivity analyses were performed to ensure the robustness of the findings. Gene pleiotropy was assessed using the MR‐Egger intercept test, with *p* > 0.05 indicating no significant pleiotropy. Furthermore, scatter plots were employed to examine the influence of outliers on the causal relationship, and Cochrane’s Q‐tests were used to assess heterogeneity across single nucleotide polymorphisms (SNPs), with *p* > 0.05 indicating the absence of heterogeneity. Funnel plots were also used to further validate the robustness of the causal inference and to detect potential outliers. The impact of individual SNPs on the overall MR estimate was evaluated through leave‐one‐out analysis.

### 2.3. Intermediary Analysis

In this two‐sample MR study, we identified an increased risk of IPF associated with COVID‐19. To investigate potential mediators in the pathway by which COVID‐19 promotes IPF, we conducted a comprehensive evaluation of 1,400 plasma metabolites (Figure 1). The mediating effects were quantified using a two‐step MR approach: mediating effect = *β* (*A*) × *β* (*B*). The direct effect was calculated as the difference between the total effect and the mediated effect (i.e., direct effect = total effect—mediated effect). The proportion of the mediated effect was then calculated using the formula: mediated effect proportion = (mediated effect/total effect) × 100%. The 95% CI for the mediated effect proportion was estimated using the delta method. Mediators were considered to have strong evidence if they simultaneously met the criteria for exposure‐to‐outcome, mediator‐to‐outcome, and exposure‐to‐mediator associations, and if their mediation effect was significantly different from zero.

### 2.4. Tool Variable Selection

In this study, instrumental variable (IV) selection strictly adhered to the three fundamental assumptions of IVs: (1) IVs must be significantly associated with the exposure factors; (2) IVs must not be related to any confounding factors; and (3) IVs must affect the outcome variables only through the exposure factors. Specifically, the selected IVs met the following criteria: (1) In the original GWAS, when genome‐wide significant loci were insufficient, SNPs with *p* < 5 × 10^6^, *p* < 5 × 10^7^, or those reaching the genome‐wide significance threshold of *p* < 5 × 10^−8^ were considered potential IVs; (2) SNPs significantly associated with the outcome variables (*p* < 0.05) were excluded to avoid direct associations with the outcome; (3) the influence of linkage disequilibrium was minimized by clustering (*r* ^2^ < 0.3, window size = 1000 kb; *r* ^2^ < 0.01, window size = 500 kb; or *r* ^2^ < 0.001, window size = 10,000 kb); (4) the accuracy of MR analysis was ensured by detecting and removing pleiotropy using the MR‐PRESSO method; and (5) the strength of the selected SNPs was assessed using *F*‐statistics, excluding SNPs with *F*‐statistics < 10 to prevent weak instrument bias. This rigorous IV selection process ensured the reliability of the MR analysis.

### 2.5. scRNA‐seq Analysis Process

#### 2.5.1. scRNA Data Processing

We used the Seurat package (v4.0) to analyze single‐cell and single‐nucleus RNA sequencing data from lung tissues of COVID‐19 patients. The analysis included several key steps: data quality control to remove low‐quality cells (fewer than 200 or more than 5000 genes, or more than 20% mitochondrial genes), data normalization and standardization (LogNormalize, FindVariableFeatures, ScaleData), sample integration and batch effect removal using Harmony, and dimensionality reduction with PCA, followed by clustering and UMAP for visualization. Cell types were identified using marker genes and tools like clustermole and singscore.

#### 2.5.2. Identification and Functional Analysis of Differentially Expressed Genes (DEGs)

DEGs between different cell subpopulations or disease states were identified using the FindMarkers function, with *p* < 0.05 and avg_log2FC > 0.25 considered statistically significant. Functional analysis of these DEGs was performed using the clusterProfiler package to conduct GO and KEGG enrichment analysis, helping to clarify their roles in biological functions. Additionally, gene set scoring was done using the AddModuleScore function in Seurat, with scores displayed on a color scale from purple (high) to gray (low).

#### 2.5.3. Analysis of Intercellular Communication

Intercellular communication and the orchestration of cellular functions were explored using the CellChat tool, providing insights into how different cell types interact and coordinate within the tissue environment. This comprehensive analysis allowed for the detailed characterization of cellular responses and interactions in the context of COVID‐19.

#### 2.5.4. Cell Culture

The SARS‐CoV‐2 plasmid was purchased from Hunan Fenghui Biotechnology (Hunan, China). Lentiviral particles were generated by co‐transfecting SARS‐CoV‐2, psPAX2, and pMD2G into 293T cells (ATCC) using Lipofectamine transfection. Viral supernatants were collected and filtered. After transducing siNC, siSlc16a4, and siPdgfra into MLE12 cells (ATCC) for 12 h, the cells were treated with SARS‐CoV‐2 virus and cocultured with 3T3 cells for 48 h. Then, the MLE12 cells, their culture supernatant, and 3T3 cells (ATCC) were collected for further analysis.

#### 2.5.5. Quantitative PCR

Total RNA was isolated using the TRIzol reagent (15596‐026, Life Technologies, Carlsbad, CA, USA), and reverse transcription was processed with reverse transcription kit under the guidance of the manual. qPCR assay was processed using multiple kits (ChamQ Universal SYBR qPCR Master Mix) (Q711‐02, Vazyme) in accordance with the manufacturer’s instructions. Relative expression level of mRNA was determined by normalizing each gene’s expression level to that of the internal reference gene GAPDH, and the primer sequences used in this experiment are shown in Tables 1 and 2.

**Table 1:** Primer sequences used for PCR.

Primer name	Sequence
TGF‐β—forward	CCA CCT GCA AGA CCA TCG AC
TGF‐β—reverse	CTG GCG AGC CTT AGT TTG GAC
COL1A—forward	GGT​GTG​AAC​TGT​CAC​CGA​TCA
COL1A—reverse	GTT​TAG​GAT​GTG​AAC​CTC​CCT​TG
GAPDH—forward	GAT GGG TGT GAA CCA CGA GA
GAPDH—reverse	CAG ATC CAC GAC GGA CAC AT
α‐SMA—forward	CCC​AGA​CAT​CAG​GGA​GTA​ATG​G
α‐SMA—reverse	TCT​ATC​GGA​TAC​TTC​AGC​GTC​A
Slc16a4—forward	GCA​CCT​GCT​ATA​TCC​TCT​CTT​CA
Slc16a4—reverse	CGT​TTC​CAT​CTT​TCA​GCC​AGT​G
Pdgfra—forward	AGA​GTT​ACA​CGT​TTG​AGC​TGT​C
Pdgfra—reverse	GTC​CCT​CCA​CGG​TAC​TCC​T

**Table 2:** siRNA primer sequences used in the experiment.

Gene	Sequence 5′ ⟶ 3′
si‐mus‐Pdgfra—forward	GAU​GAU​CUG​CAA​GCA​UAU​UAA​TT
si‐mus‐Pdgfra—reverse	UUA​AUA​UGC​UUG​CAG​AUC​AUC​TT
si‐mus‐Slc16a4—forward	CCA​CAG​CCA​AUC​AAA​CAA​CAA​TT
si‐mus‐Slc16a4—reverse	UUG​UUG​UUU​GAU​UGG​CUG​UGG​TT

#### 2.5.6. Western Blot

Isolated cells or tissues were placed on ice, and proteins were extracted with RIPA buffer (Millipore) plus protease inhibitor cocktail and phosphatase inhibitor cocktail (MCE). The protein concentration was determined with the BCA protein assay kit (Thermo Fisher Scientific). 30–60 μg protein was loaded per well on 6%–12% SDS‐PAGE gel and transferred onto PVDF membrane (Thermo Fisher Scientific), treated by ethanol. Membranes were washed with 1 × TBST (1 × TBS with 0.1% Tween‐20), blocked with 5% skim milk, and incubated with antibodies against α‐SMA (Proteintech, 14395‐1‐AP), or GAPDH (Servicebio, GB11002), followed by incubation with the HRP‐linked secondary antibody for 1 h at room temperature, and the membrane was developed with ECL Prime Western Blotting Detection Reagent.

### 2.6. Statistical Analysis

All data were presented as mean ± standard error (SE). Group comparisons were performed using *t*‐tests for two groups and one‐way ANOVA for comparisons involving more than two groups. All calculations and analyses were conducted using either Prism 9 software (GraphPad) or the R programming language. DEGs within each cluster were identified using the Wilcoxon rank‐sum test. Pearson’s correlation analysis was employed to assess relationships between two variables, with statistical significance defined as *p* < 0.05.

## 3. Results

### 3.1. Causal Impact of COVID‐19 on IPF

The GWAS for COVID‐19 included 1,388,342 individuals, while the study for IPF involved 409,798 individuals. MR analysis revealed a significant positive association between COVID‐19 infection and an increased risk of developing IPF (OR = 1.15, 95% CI = 1.05–1.25, *p* = 0.001). Sensitivity analyses confirmed the robustness of these findings, with *q*‐statistics indicating no evidence of heterogeneity. Furthermore, MR‐Egger regression and MR‐PRESSO analyses found no evidence of horizontal pleiotropy (Figure 2(a)). To exclude the possibility of reverse causality, a reverse MR analysis was conducted, which demonstrated no causal effect of IPF on COVID‐19 infection (Figure 2(b)). In conclusion, the two‐sample MR analysis identified COVID‐19 as a risk factor for IPF.

Figure 2Causal relationship between COVID‐19 and IPF (a) The effect of COVID‐19 on idiopathic pulmonary fibrosis (IPF) was assessed using inverse variance weighting (IVW), MR‐Egger, and weighted median methods across all major Mendelian randomization (MR) analyses. *p*‐value < 0.05 for IVW (adjusted using Benjamini–Hochberg multiple‐test correction), along with directionally consistent results from MR‐Egger and weighted median analyses, was considered strong evidence of causality. MR: Mendelian randomization. (b) Reverse Mendelian randomization analysis demonstrated that IPF does not causally influence the risk of COVID‐19 infection.

**Figure:**
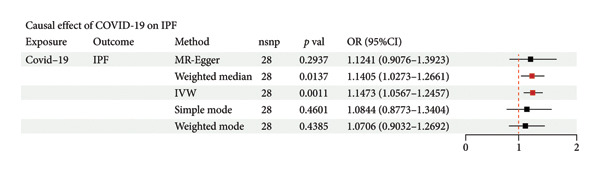
(a)

**Figure:**
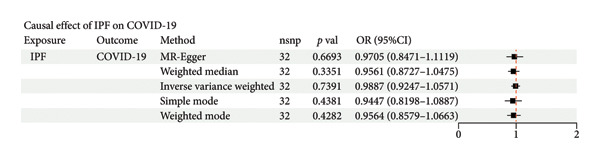
(b)

### 3.2. COVID‐19 Promotes IPF Through Lactate Metabolism

To investigate the potential mediating mechanisms underlying the causal relationship between COVID‐19 and IPF, we conducted a two‐step MR mediation analysis. In the first step, we evaluated 1,400 plasma metabolites as exposure factors, with IPF as the outcome, and identified 31 plasma metabolites as risk factors for IPF (Figure 3(a)). Sensitivity analyses confirmed that there was no significant heterogeneity or horizontal pleiotropy in these MR analyses, ensuring the robustness of the findings. In the second step, two‐sample MR was performed to assess the causal effect of COVID‐19 on these 31 plasma metabolites linked to IPF. Using COVID‐19 as the exposure and the selected plasma metabolites as the outcome, we identified a significant causal relationship between COVID‐19 and lactate metabolism (OR = 1.037, 95% CI = 1.00–1.07, *p* = 0.04). The results were consistent in terms of overall, indirect, and direct effects, and leave‐one‐out analyses further supported the reliability of the causal relationship in this exposure–mediator–outcome model (Figure 3(b)). These findings suggest that lactate metabolism may play a key role in mediating the impact of COVID‐19 on IPF.

Figure 3Two‐step MR analysis of lactate metabolism as a mediating mechanism in the causal relationship between COVID‐19 and IPF (a) The causal effect of plasma metabolites on idiopathic pulmonary fibrosis (IPF). (b) The causal effect of COVID‐19 on plasma metabolites. A *p*‐value < 0.05 for inverse variance weighting (IVW) analysis (adjusted using Benjamini–Hochberg multiple‐test correction), along with directionally consistent results from MR‐Egger and weighted median analyses, was considered strong evidence of causality.

**Figure:**
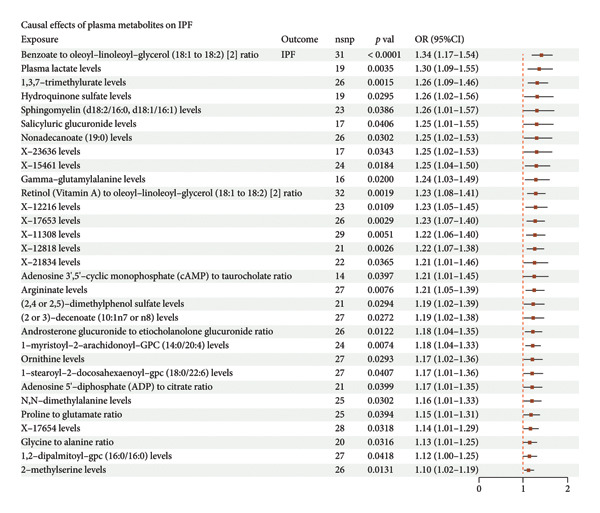
(a)

**Figure:**
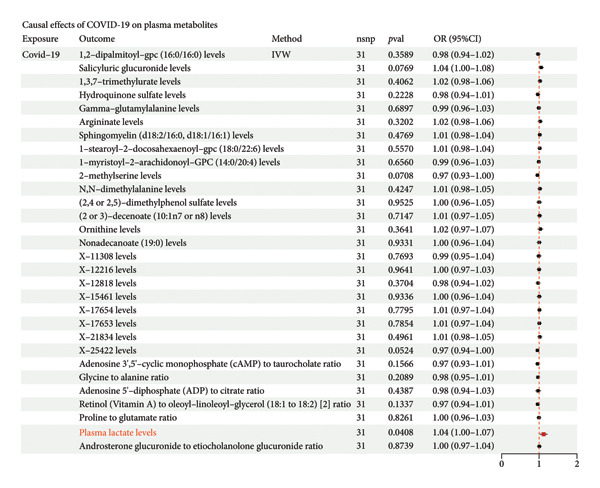
(b)

### 3.3. AT1 Cell–Associated Lactate Metabolism Links COVID‐19 to Fibroblast Activation and Fibrosis

To investigate the biological role of lactate in the lungs of patients infected with SARS‐CoV‐2, we analyzed scRNA‐seq data from lung tissues of both COVID‐19 patients and healthy controls. Through cellular subpopulation clustering and marker gene analysis, we identified 15 distinct cell populations, including vascular endothelial cells, macrophages, neutrophils, lymphatic endothelial cells, fibroblasts, Type II alveolar epithelial cells, T‐lymphocytes, Type I alveolar epithelial cells, B‐lymphocytes, dendritic cells, cuprocytes, smooth muscle cells/pericytes, mast cells, and monocytes (Figure 4(a)). Next, an intersection analysis was performed between lactate‐related genes and DEGs in COVID‐19 patients, revealing 18 overlapping genes: GCDH, TGFB2, SHC3, GSR, PIK3C2A, HTT, DNM1L, HIF1A, PTP4A2, ADRB2, PDP1, SLC16A4, FOXM1, P4HB, MMP2, DISC1, CAV1, and SPAG6 (Figure 4(b)). UMAP downscaling and gene set scoring (AddModuleScore) showed that the expression of lactate metabolism‐related genes was significantly higher in the lung tissues of COVID‐19 patients compared to healthy controls (*p* < 0.0001) (Figures 4(c) and 4(d)). Moreover, the gene set related to lactate metabolism was significantly positively correlated with the expression of fibroblast marker genes in lung tissues (*R* = 0.76, *p* < 0.0001) (Figure 4(e)), suggesting that lactate metabolism may contribute to fibrosis during COVID‐19 infection. Cell type–specific analysis revealed that lactate metabolism gene set scores were elevated in multiple cell types, with the most pronounced increase observed in AT1 cells (Figure 4(f)). In AT1 cells, lactate metabolism scores correlated significantly with fibroblast marker gene set scores (*R* = 0.26, *p* = 0.0089; Figure 4(g)). Similarly, SLC16A4 expression in AT1 cells also exhibited a significant positive correlation with fibroblast marker gene scores (*R* = 0.20, *p* = 0.043; Figure 4(h)).

Figure 4COVID‐19 may regulate lactate metabolism via SLC16A4 in lung epithelial cells to promote lung fibrosis (a) UMAP plot showing the clustering analysis of lung tissue cells from COVID‐19 patients and healthy controls, identifying 15 distinct cell subpopulations. (b) Intersection analysis of differentially expressed genes (DEGs) and lactate metabolism‐related gene sets in COVID‐19 patients, revealing 18 overlapping genes. (c) UMAP plot displaying lactate metabolism‐related gene set scores across all lung tissue cells. (d) Bar graph showing the statistical comparison of lactate metabolism‐related gene set scores between COVID‐19 patients and healthy controls, with significantly higher expression levels observed in COVID‐19 patients (*p* < 0.0001). (e) Positive correlation between lactate metabolism‐related gene set expression and fibroblast marker gene expression in lung tissues. (f) Bar graph showing the statistical distribution of lactate metabolism‐related gene set scores across different lung tissue cell subpopulations. (g) Significant positive correlation between lactate metabolism gene set scores and fibroblast marker gene set scores in AT1 cells. (h) Significant positive correlation between the expression of the lactate metabolism‐regulating gene SLC16A4 and fibroblast marker gene set scores in AT1 cells. (i) The lactate levels of MLE12 cells after treating with SARS‐CoV‐2. (j, k, l) Relative α‐SMA, Tgf‐β, and Col1A mRNA expression are shown by RT‐qPCR in 3T3 cells after coculture with MLE12 cells for 48 h; MLE12 cells were treated with SARS‐CoV‐2. (m) Dot plot illustrating the expression levels of lactate metabolism‐related gene sets across each lung tissue cell subpopulation. (n) Violin plots showing SLC16A4 expression levels in AT1 cells from control and COVID‐19 lung samples. (o) Relative Slc164a mRNA expression is shown by RT‐qPCR in MLE12 cells after treating with SARS‐CoV‐2. (p) Relative Slc164a mRNA expression is shown by RT‐qPCR after knockdown of Slc164a. (q) The lactate levels of MLE12 cells after treating with SARS‐CoV‐2 and siNC or siSlc16a4. (r, s, t) Relative mRNA expression levels of α‐SMA, Col1A, and Tgf‐β in MLE12 cells treated with SARS‐CoV‐2 pseudovirus and transfected with either negative control siRNA (siNC) or siRNA targeting SLC16A4 (siSlc16a4), as determined by RT‐qPCR.

**Figure:**
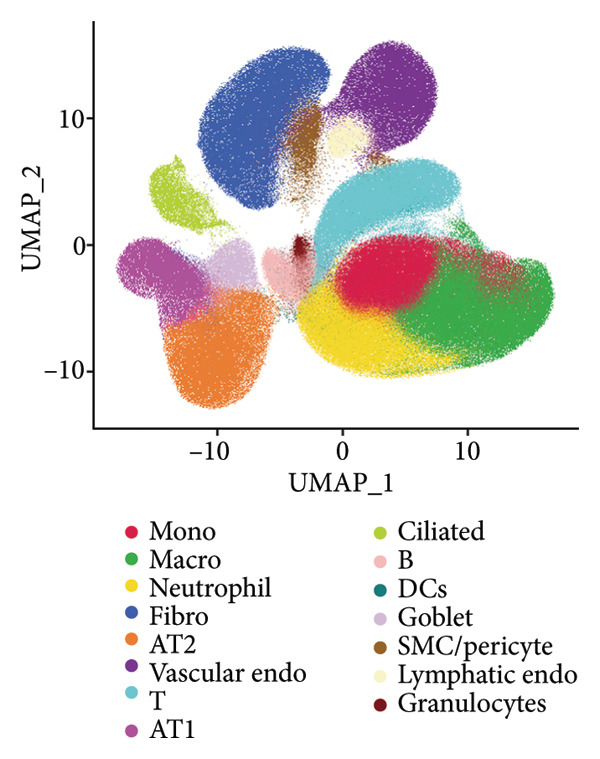
(a)

**Figure:**
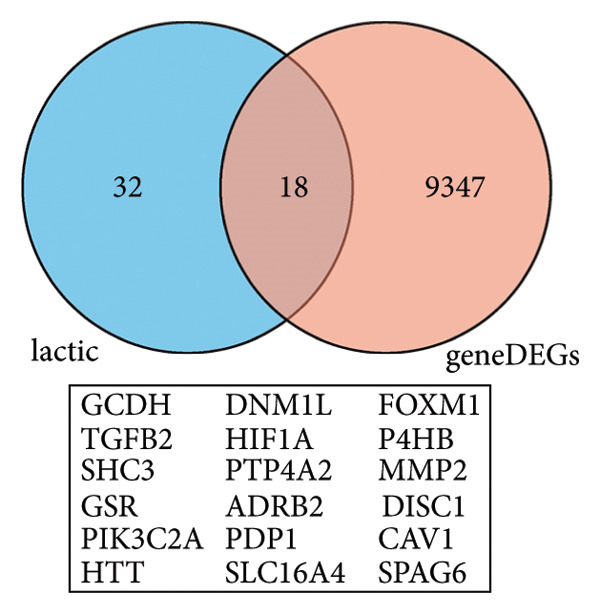
(b)

**Figure:**
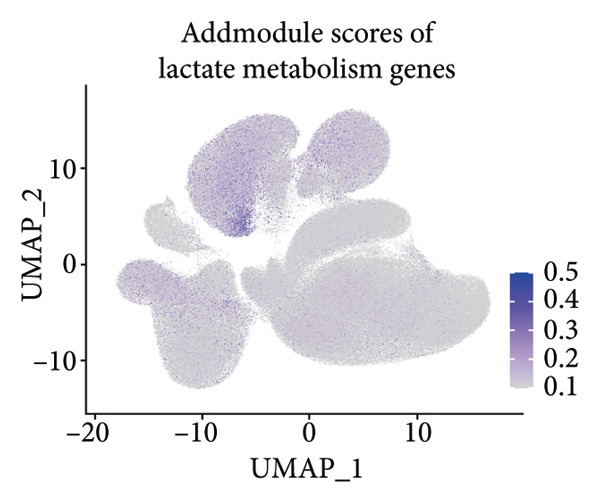
(c)

**Figure:**
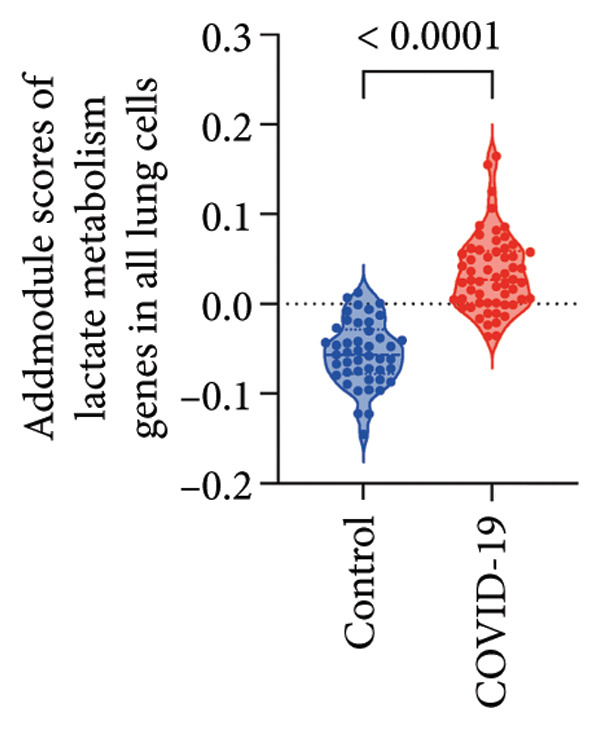
(d)

**Figure:**
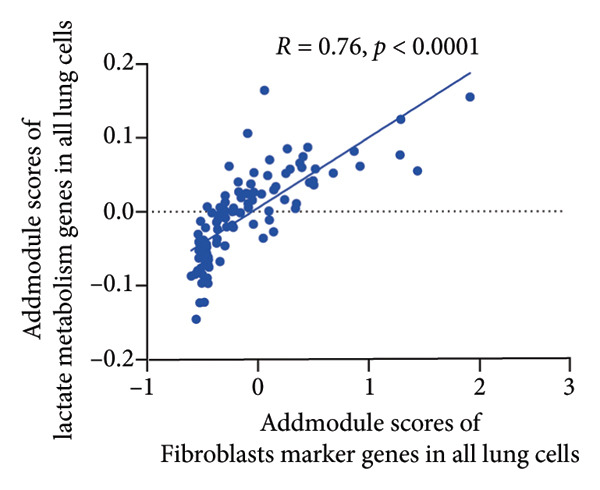
(e)

**Figure:**
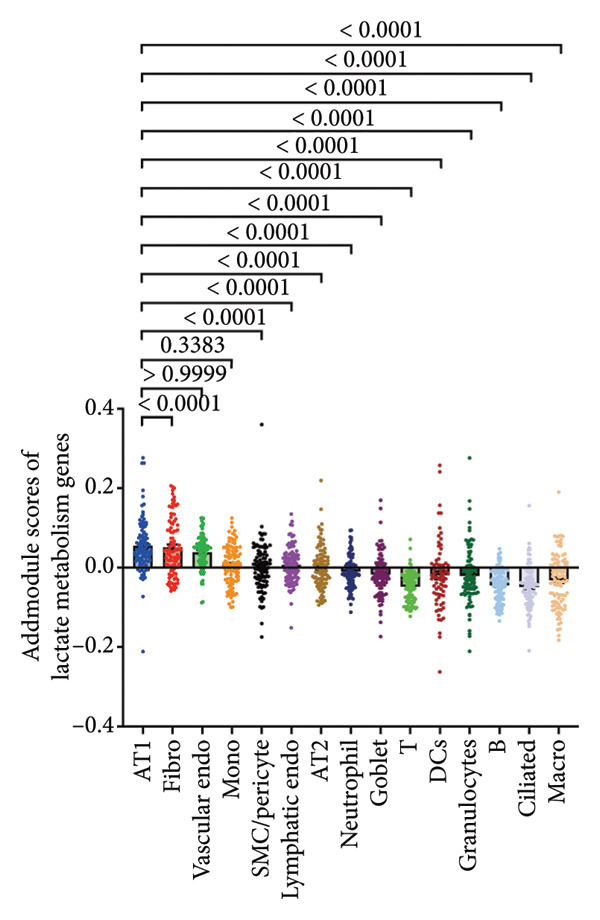
(f)

**Figure:**
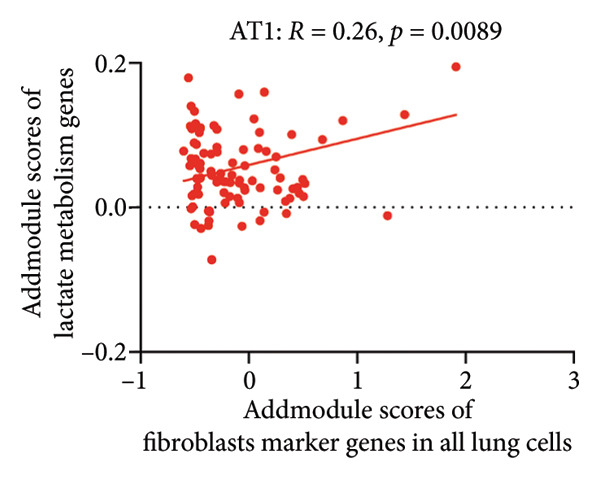
(g)

**Figure:**
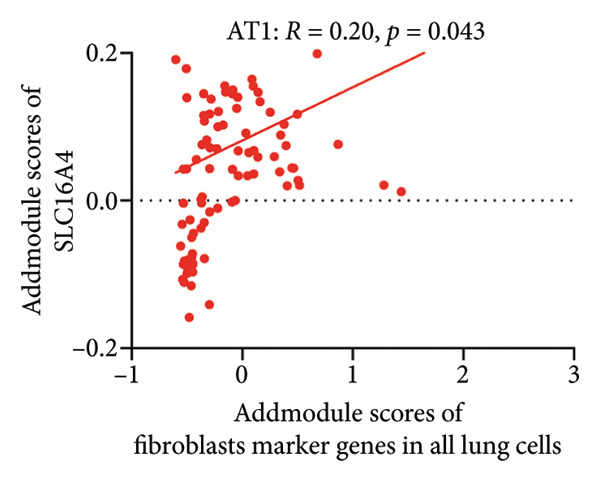
(h)

**Figure:**
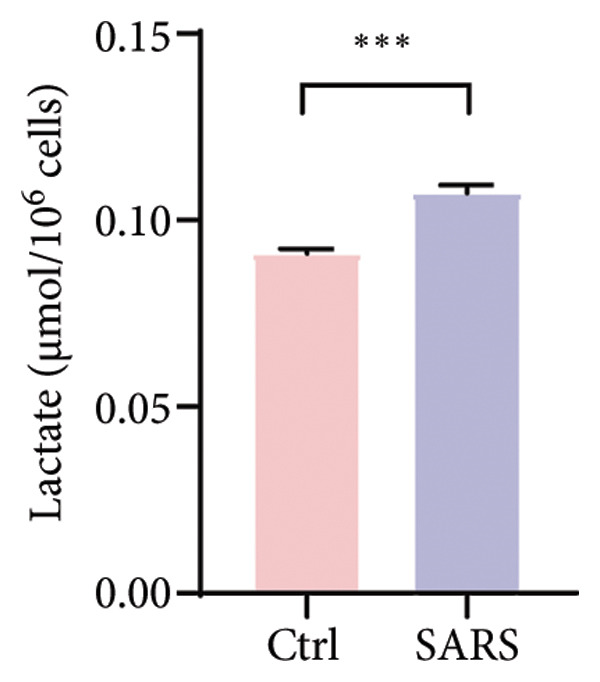
(i)

**Figure:**
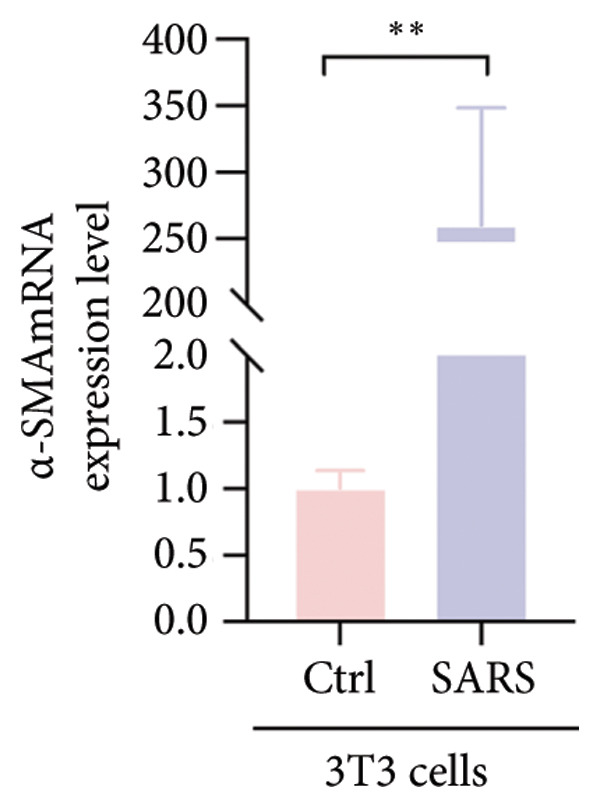
(j)

**Figure:**
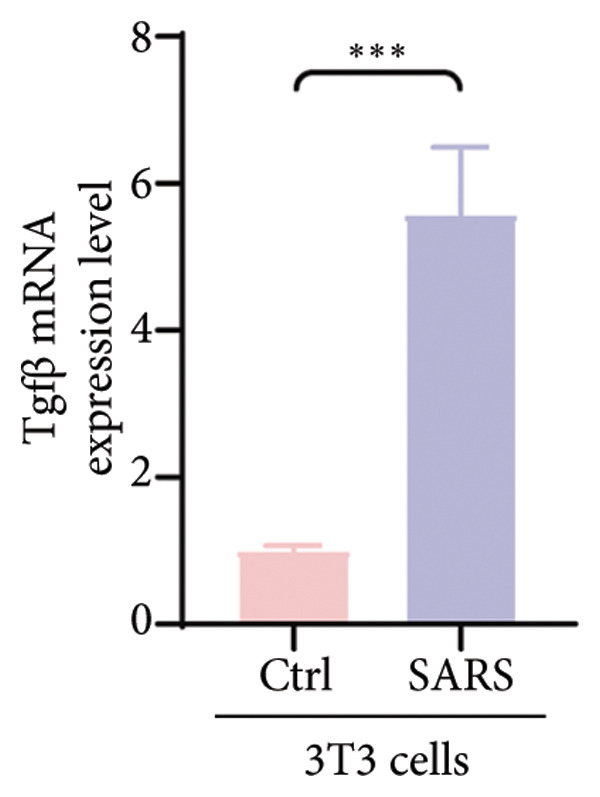
(k)

**Figure:**
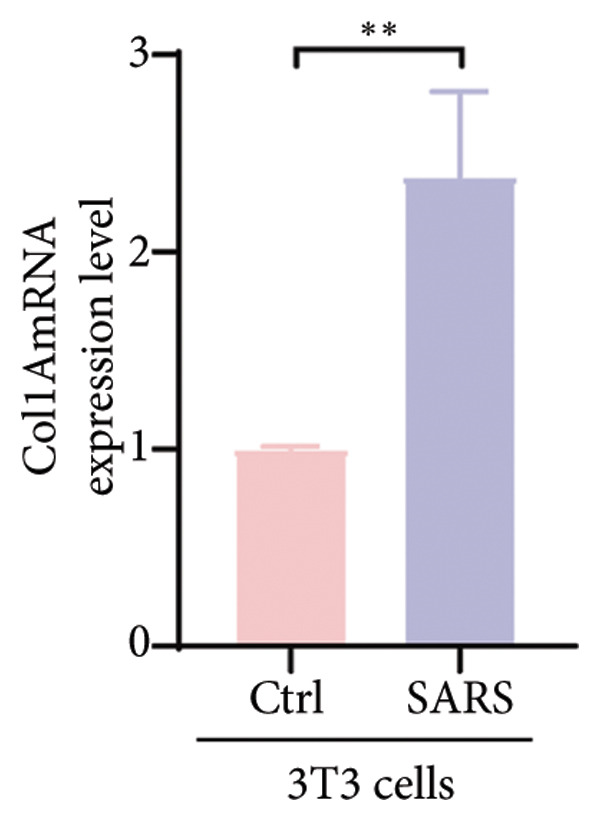
(l)

**Figure:**
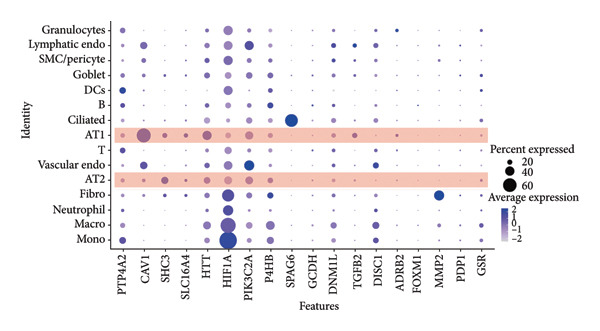
(m)

**Figure:**
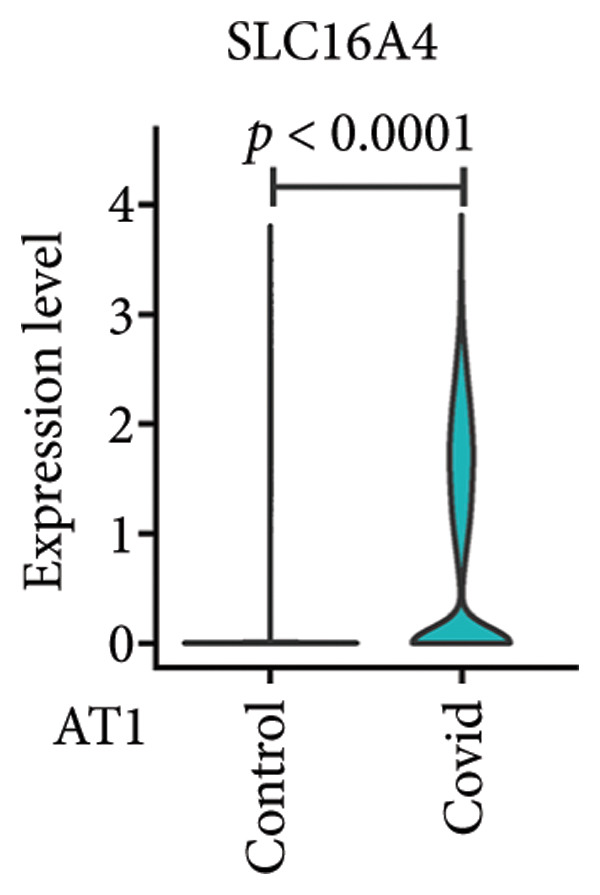
(n)

**Figure:**
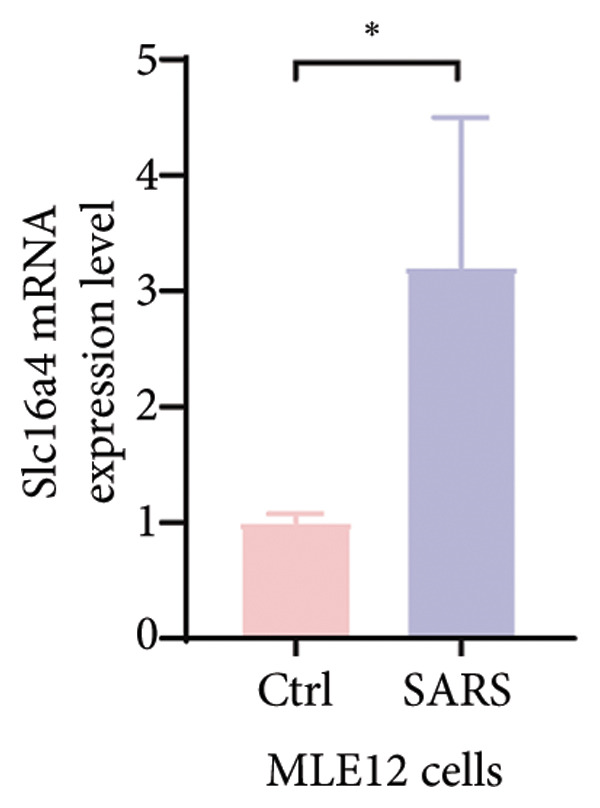
(o)

**Figure:**
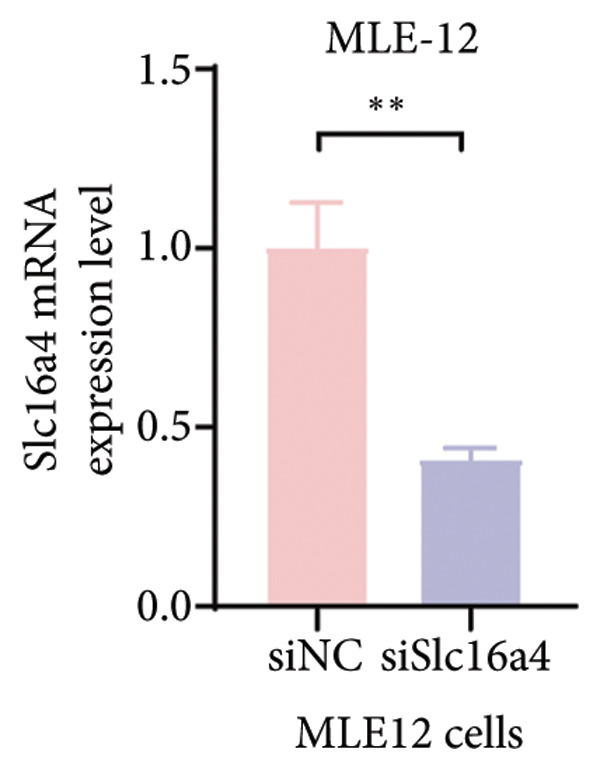
(p)

**Figure:**
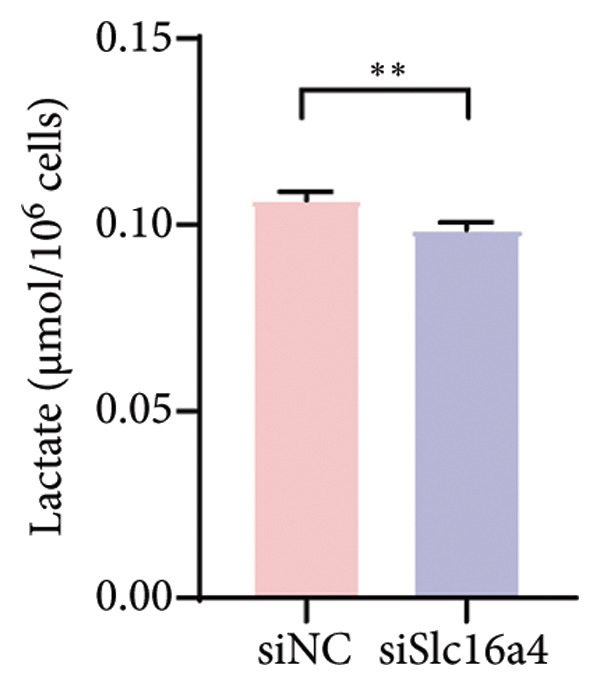
(q)

**Figure:**
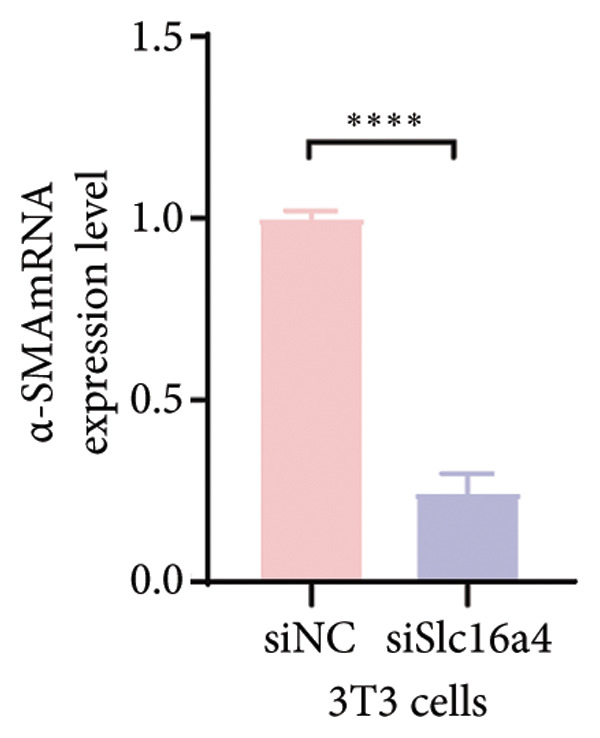
(r)

**Figure:**
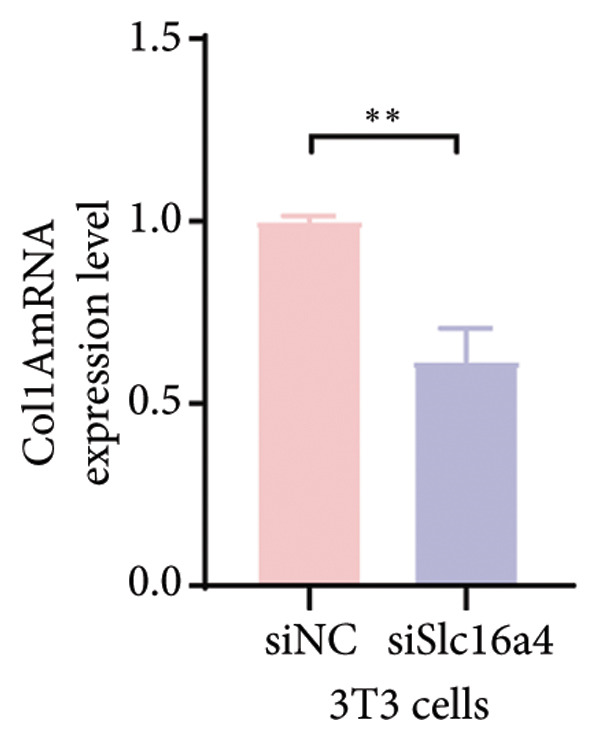
(s)

**Figure:**
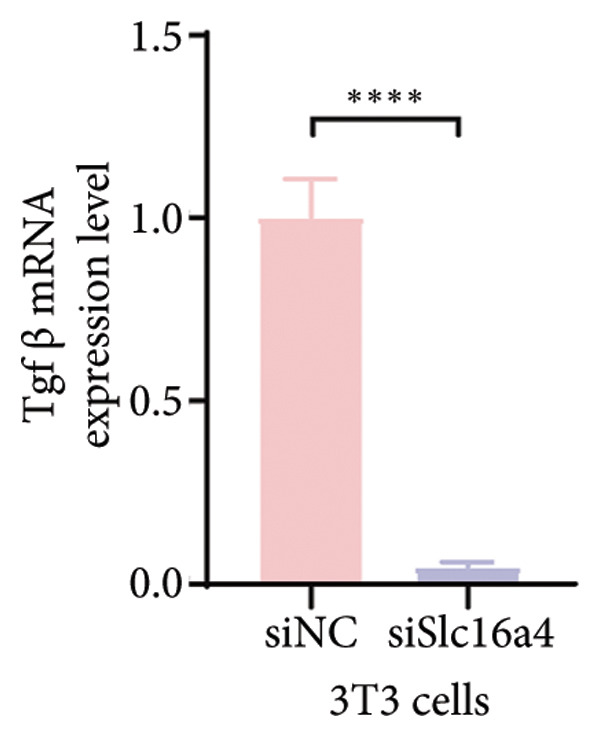
(t)

Further analysis revealed that the gene set scores for lactate metabolism were significantly and positively correlated with fibroblast marker gene set scores in both AT1 (*R* = 0.26, *p* = 0.0089) (Figure 4(h)). Additionally, the expression of SLC16A4, a key gene involved in lactate metabolism, was also significantly positively correlated with fibroblast marker gene set scores in both AT1 (*R* = 0.20, *p* = 0.043) and AT2 (*R* = 0.26, *p* = 0.0084) cells (Figure 4(i)). Dot plot analysis further confirmed broad expression of lactate metabolism–related genes across lung cell populations, with enrichment in epithelial and fibroblast compartments (Figure 4(m)). Moreover, SLC16A4 expression was markedly elevated in AT1 cells from COVID‐19 lungs compared with controls (*p* < 0.0001; Figure 4(n)).

To experimentally validate these findings, we performed in vitro coculture assays using murine alveolar epithelial MLE12 cells and 3T3 fibroblasts. SARS‐CoV‐2 exposure significantly increased lactate production in MLE12 cells (Figure 4(i)) and, in coculture, induced robust upregulation of fibrosis‐associated genes α‐SMA (Acta2), Tgf‐β, and Col1a in fibroblasts (Figures 4(j), 4(k), 4(l)). SARS‐CoV‐2 infection also elevated Slc16a4 mRNA levels in MLE12 cells (Figure 4(o)). Knockdown of Slc16a4 by siRNA (Figure 4(p)) markedly reduced lactate accumulation (Figure 4(q)) and attenuated the induction of α‐SMA, Col1a, and Tgf‐β in fibroblasts cocultured with infected MLE12 cells (Figures 4(r), 4(s), 4(t)). Our results suggest that altered lactate metabolism, particularly via SLC16A4 in AT1 epithelial cells, may contribute to fibroblast activation and fibrosis in the context of COVID‐19.

### 3.4. Potential Role of PDGFC–PDGFRA Signaling in Epithelial–Fibroblast Crosstalk

Cell–cell communication analysis revealed extensive intercellular interactions in lung tissues from COVID‐19 patients and controls, with AT1 epithelial cells showing particularly frequent interactions with fibroblasts (Figures 5(a) and 5(b)). Among these, the PDGFC–PDGFRA signaling axis appeared as one of the enriched pathways mediating epithelial–fibroblast communication in COVID‐19 lungs (Figure 5(c)). In vitro coculture assays further showed that exposure of epithelial cells to SARS‐CoV‐2 pseudovirus was associated with increased Pdgfra mRNA expression in 3T3 fibroblasts (Figure 5(d)). siRNA‐mediated knockdown of Pdgfra in fibroblasts (Figure 5(e)) attenuated the upregulation of α‐SMA observed after coculture with infected epithelial cells (Figure 5(f)). These findings suggest that PDGFC–PDGFRA signaling may participate in epithelial–fibroblast crosstalk during the fibrotic response in the context of SARS‐CoV‐2 infection.

Figure 5Potential role of PDGFC–PDGFRA signaling in epithelial–fibroblast crosstalk (a) graphical representation of single‐cell RNA sequencing data from lung tissue analyzed using CellChat, illustrating the strength and number of interactions between various cell types. (b) Quantification of the strength and number of interactions between AT1 epithelial cells and other cell types, with particularly strong interactions observed between epithelial cells and fibroblasts. (c) Scatterplots showing the interactions of AT1 epithelial cells with other cell subpopulations in both healthy controls and COVID‐19 patients. The PDGFC–PDGFRA signaling axis was identified as a significant interaction between epithelial cells and fibroblasts, indicating its key role in the regulation of fibroblast activity in COVID‐19‐associated lung fibrosis. (d) Relative Pdgfra mRNA expression is shown by RT‐qPCR in 3T3 cells after coculture with MLE12 cells for 48 h; MLE12 cells were treated with SARS‐CoV‐2. (e) Knockdown efficiency of Pdgfra in 3T3 cells using siRNA. (f) Relative α‐SMA mRNA expression is shown by RT‐qPCR in 3T3 cells after coculture with MLE12 cells for 48 h; MLE12 cells were treated with SARS‐CoV‐2 and siNC or Pdgfra.

**Figure:**
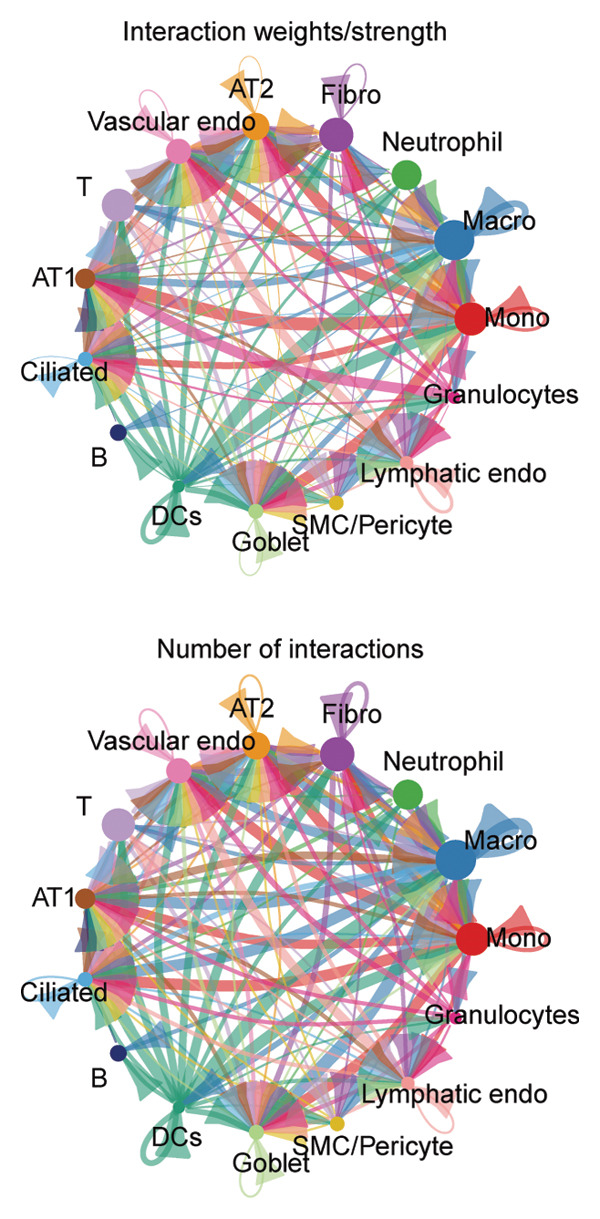
(a)

**Figure:**
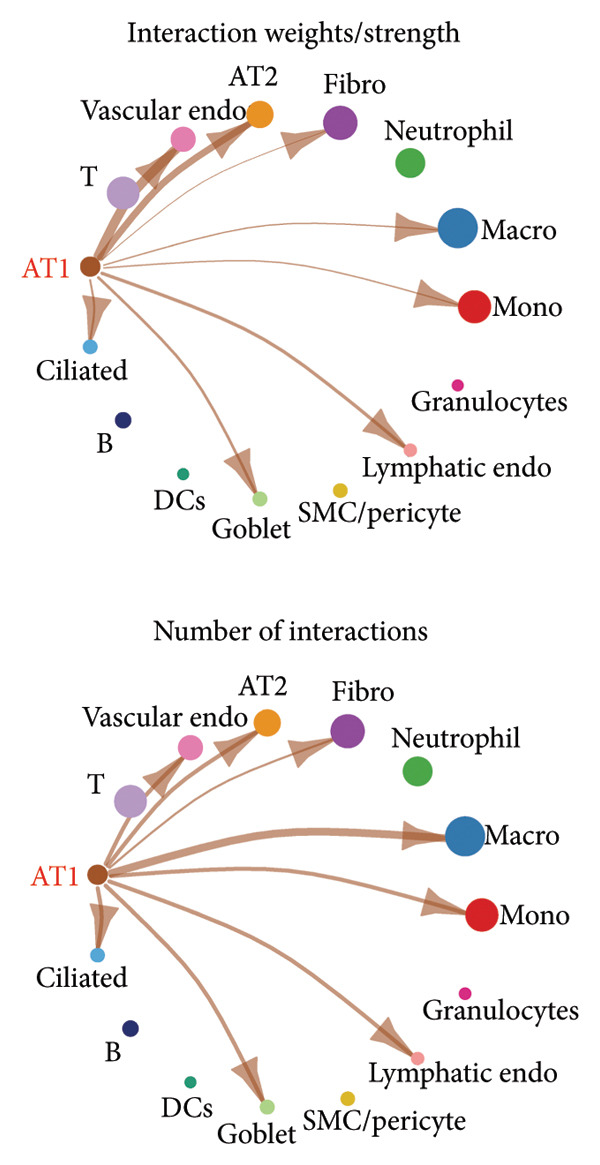
(b)

**Figure:**
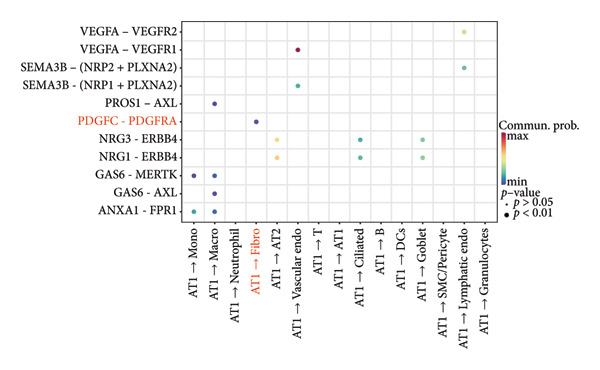
(c)

**Figure:**
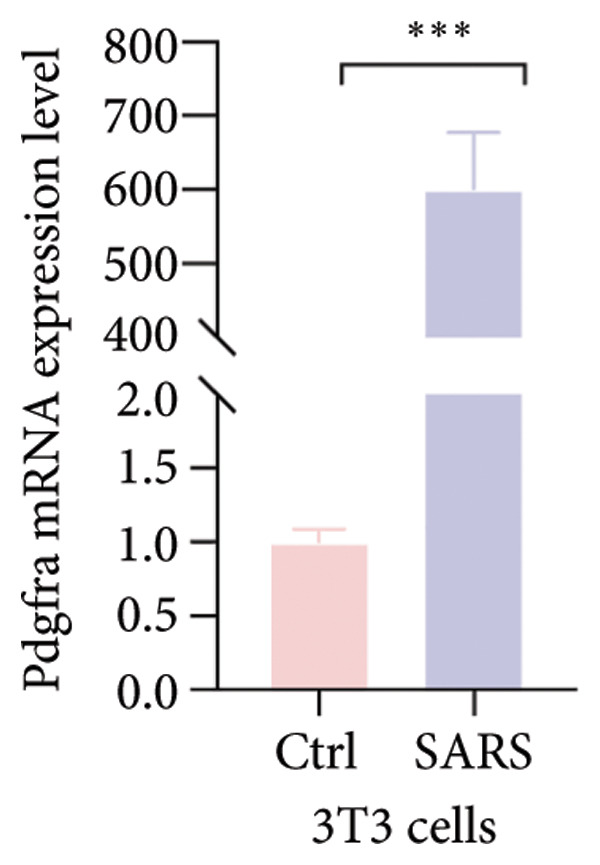
(d)

**Figure:**
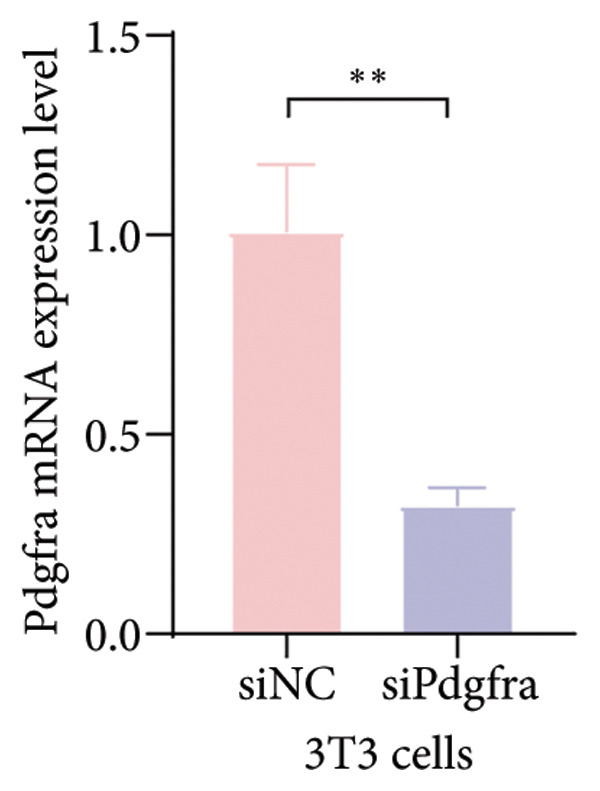
(e)

**Figure:**
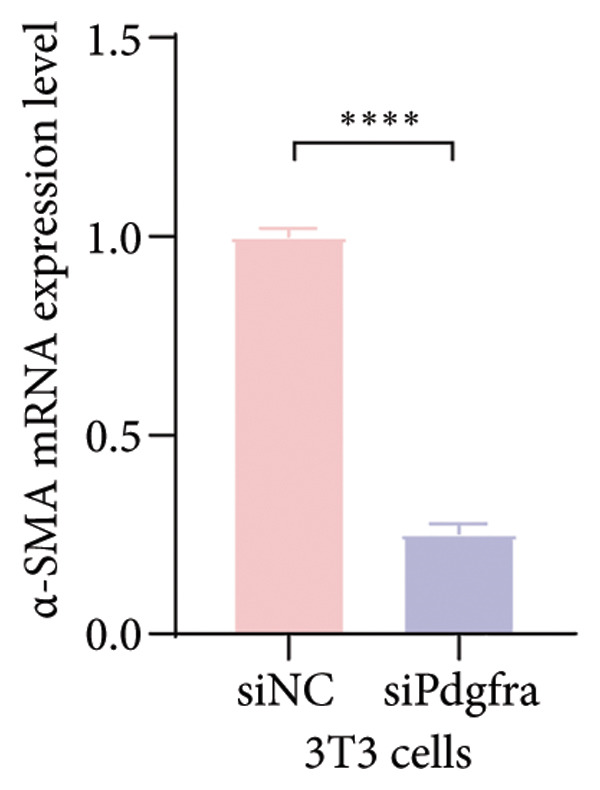
(f)

## 4. Discussion

This study reveals that COVID‐19 promotes the development of pulmonary fibrosis through the lactate metabolic pathway regulated by the SLC16A4 gene in lung epithelial cells. By integrating MR analysis, scRNA‐seq, and targeted in vitro assays, our study provides mechanistic insights into how COVID‐19 drives fibrotic remodeling, while also identifying potential molecular targets for therapeutic intervention.

### 4.1. Causal Effect of COVID‐19 on IPF

Our study found that COVID‐19 infection was a causal factor in the development of IPF, whereas IPF was not significantly promoted by COVID‐19 infection. COVID‐19 contributes to the development and progression of IPF through multiple profibrotic pathways, such as the TGF‐β signaling pathway. Following COVID‐19 infection, lung epithelial cell injury and subsequent repair processes are often accompanied by aberrant fibroblast activation and excessive deposition of ECM, ultimately leading to fibrosis [18, 19]. It has been shown that patients with IPF may be at higher risk and have more severe clinical outcomes after COVID‐19 infection due to impaired lung function, abnormal immune responses, and other factors [20, 21]. However, our study drew different conclusions, and through Mendelian randomization analysis, we found that IPF did not significantly increase the risk of COVID‐19 infection. This difference may stem from differences in study design and methodology. Most of the previous studies were retrospective cohort studies, which may be affected by confounding factors, such as age, comorbidities, and decreased lung function, which may have masked the true causal relationship between IPF itself and COVID‐19 infection. Our study used Mendelian randomization analysis, a method that better avoids the influence of confounding factors and reveals the true causal relationship between COVID‐19 and IPF. In addition, previous studies have focused on the clinical outcomes of IPF patients after COVID‐19 infection, whereas our study focuses more on how COVID‐19 drives the onset and progression of IPF through molecular pathways, which provides a different research perspective.

### 4.2. COVID‐19 Promotion of IPF Through Lactate Metabolism

Our mediation analysis further highlights lactate metabolism as a key pathway through which COVID‐19 promotes IPF. scRNA‐seq confirmed the central role of lactate metabolism in pulmonary fibrosis, particularly in lung epithelial cells, where lactate metabolism genes exhibited significant regulatory functions. Disruptions in lactate metabolism not only impact lung epithelial cells at the metabolic level but also contribute to the initiation and progression of fibrosis by influencing intercellular signaling and the tissue microenvironment. Lactate has been shown to play a crucial profibrotic role in pulmonary fibrosis. Beyond its function as a metabolic by‐product, lactate acts as a signaling molecule that exacerbates fibrosis by altering the local microenvironment. This process activates fibroblasts and promotes ECM deposition [22]. Specifically, lactate enhances the fibrotic microenvironment by modulating immunosuppressive cells, such as regulatory *T* cells (Tregs), and inducing the secretion of proinflammatory cytokines like IL‐6 and IL‐1β [23]. COVID‐19 infection amplifies these effects, particularly in severe cases where lung tissue exhibits significantly elevated lactate levels. This is accompanied by the activation of multiple profibrotic signaling pathways, including TGF‐β, which accelerates the development of pulmonary fibrosis [24].

Our in vitro experiments further supported these findings. SARS‐CoV‐2 pseudovirus exposure significantly increased lactate production in MLE12 alveolar epithelial cells, and fibroblasts cocultured with infected epithelial cells exhibited robust upregulation of fibrosis‐associated genes (α‐SMA, Col1a, and Tgf‐β). Knockdown of Slc16a4, a lactate transporter upregulated in AT1 cells, reduced epithelial lactate accumulation and attenuated fibroblast activation. These results provide experimental evidence linking epithelial lactate metabolism to fibroblast profibrotic responses, consistent with prior observations that metabolic reprogramming in epithelial cells contributes to pathological lung remodeling [25].

### 4.3. SLC16A4 Regulates Lactate Metabolism in COVID‐19‐Induced Pulmonary Fibrosis

Monocarboxylate transporters (MCTs) of the SLC16 family are critical for transmembrane lactate flux [26, 27]. Our study shows that COVID‐19 infection upregulates SLC16A4 expression in lung epithelial cells, leading to lactate accumulation, which in turn promotes the progression of pulmonary fibrosis. In vitro, siRNA‐mediated knockdown of Slc16a4 reduced lactate levels and downstream fibroblast activation, supporting its role as a mediator of epithelial–fibroblast crosstalk.

Previous work has shown that SLC16A4 regulates lactate transport across endothelial barriers in the central nervous system [28]. Similarly, in COVID‐19‐associated pulmonary fibrosis, SLC16A4 may mediate intercellular lactate signaling, activating fibrosis‐related pathways, such as fibroblast proliferation and ECM accumulation. This study highlights the critical role of SLC16A4 in regulating lactate metabolism during COVID‐19 infection and suggests that SLC16A4 may be a potential therapeutic target for preventing or treating lung fibrosis.

### 4.4. COVID‐19 Interacts With Fibroblasts Through the PDGFC–PDGFRA Signaling Axis

In addition to metabolic reprogramming, our CellChat analysis revealed that COVID‐19 strengthens epithelial–fibroblast communication via the PDGFC–PDGFRA signaling axis. The development of organ fibrosis involves multiple signaling pathways, with the platelet‐derived growth factor (PDGF) pathway being a key mediator [29]. The PDGF family consists of disulfide‐bonded homo‐ and heterodimers of four subunits (PDGF‐A, PDGF‐B, PDGF‐C, and PDGF‐D), which exert their effects by binding to homodimeric or heterodimeric forms of two receptor proteins, PDGFR‐alpha and PDGFR‐beta, activating the receptor’s tyrosine kinase activity [30]. In vitro, SARS‐CoV‐2 exposure increased Pdgfra mRNA in fibroblasts, and siRNA knockdown of Pdgfra attenuated α‐SMA induction at both mRNA and protein levels. Interestingly, Pdgfra silencing also reduced lactate accumulation in epithelial culture supernatants, suggesting a functional link between PDGF signaling and metabolic remodeling. Previous research has also shown that the PDGF‐PDGFR signaling system is implicated in IPF, as well as in fibrosis induced by asbestos, bleomycin, and radiation [31–33]. Additionally, this pathway has been linked to fibrosis in other organs, including the kidneys, liver, skin, and heart [34, 35]. Therefore, inhibitors targeting this signaling pathway may play an important role in the treatment of COVID‐19‐associated pulmonary fibrosis.

### 4.5. Clinical Application Outlook

Given the pivotal role of SLC16A4 and its regulation of lactate metabolism in COVID‐19‐associated pulmonary fibrosis, targeting this pathway presents promising therapeutic potential. Inhibiting SLC16A4 could block lactate transport, potentially slowing fibrosis progression. Additionally, antifibrotic agents like nintedanib, which inhibits PDGFR activity, have shown efficacy in treating IPF. This suggests that targeting the PDGFC–PDGFRA signaling axis may also be a viable strategy for managing COVID‐19‐induced fibrosis. However, our study is limited to scRNA‐seq data mainly from severe cases and in vitro assays, lacking in vivo or clinical validation. Future work with animal models and longitudinal patient cohorts is needed to further confirm the role of lactate metabolism and assess the therapeutic value of targeting SLC16A4 and PDGFRA pathways.

## 5. Conclusion

In summary, our findings highlight lactate metabolism, particularly via SLC16A4 and PDGFC–PDGFRA signaling, as a potential driver of COVID‐19–associated pulmonary fibrosis and a promising target for future therapeutic intervention.

## Disclosure

All authors read and approved the manuscript before submission. All authors consented to publication.

## Conflicts of Interest

The authors declare no conflicts of interest.

## Author Contributions

Xin Zhang, Xia Tong, and Lanlan Zhang: thesis design, thesis writing, and revision; Xia Tong, Miaomiao Chen, Shuyan Yang, and Yan Mo: literature search, data compilation, raw letter analysis, thesis writing, and statistical analysis; Liping Jia and Tuersun Yeziya: performed the experiments and analyzed the data. Xin Zhang, Liping Jia, Tuersun Yeziya, and Lanlan Zhang have contributed equally to this work.

All authors were involved in the first draft of the article, and the manuscript was critically revised for rigorous and accurate intellectual content.

## Funding

The study was supported by the Technology Cooperation Project of Chengdu (2023‐GH02‐00092‐HZ), the Sichuan Medical Association Youth Innovation Project (Q21018), the Medical Research Project of Chengdu Municipal Health Commission (2022516; 2023666), and the Central Nervous System Drug Key Laboratory of Sichuan Province (230005‐01SZ).

## Data Availability

The data that support the findings of this study are available from the corresponding author upon reasonable request.
