# Correction to “Integrated Mendelian Randomization and Single‐Cell RNA Sequencing Analyses Reveal Lactate Metabolism as a Key Pathway in COVID‐19‐Induced Pulmonary Fibrosis”

**DOI:** 10.1155/carj/9867178

**Published:** 2026-09-30

X. Zhang, L. Jia, T. Yeziya, et al., “Integrated Mendelian Randomization and Single‐Cell RNA Sequencing Analyses Reveal Lactate Metabolism as a Key Pathway in COVID‐19‐Induced Pulmonary Fibrosis,” *Canadian Respiratory Journal 2026*, no. 1 (2026): 1–16, https://doi.org/10.1155/carj/1049336.

In the article titled “Integrated Mendelian Randomization and Single‐Cell RNA Sequencing Analyses Reveal Lactate Metabolism as a Key Pathway in COVID‐19‐Induced Pulmonary Fibrosis,” the authors wish to correct the article with the clarification that Liping Jia, Tuersun Yeziya, and Xin Zhang equally contributed to this study as joint first authors.

We apologize for this error.

